# The delta neutrophil index is a prognostic factor for postoperative mortality in patients with sepsis caused by peritonitis

**DOI:** 10.1371/journal.pone.0182325

**Published:** 2017-08-01

**Authors:** Jong Wan Kim, Jun Ho Park, Doo Jin Kim, Won Hyuk Choi, Jin Cheol Cheong, Jeong Yeon Kim

**Affiliations:** 1 Department of Surgery, Dongtan Sacred Heart Hospital, Hallym University, College of Medicine, Seoul, Korea; 2 Department of Surgery, Kangdong Sacred Heart Hospital, Hallym University College of Medicine, Seoul, Korea; University of Florida, UNITED STATES

## Abstract

**Introduction:**

The delta neutrophil index (DNI) represents the fraction of circulating immature granulocytes and is a marker of infection and sepsis. Our objective was to evaluate the usefulness of DNI for predicting in-hospital mortality within 30 days after surgery in patients with sepsis caused by peritonitis by means of comparing DNI, white blood cell (WBC) count, neutrophil percentage, and C-reactive protein (CRP) before and after surgery.

**Materials and methods:**

We performed a retrospective analysis of demographic, clinical, and laboratory data. DNI, WBC count, neutrophil percentage, and CRP were measured before surgery, and at 12–36 h (day 1) and 60–84 h (day 3) after surgery.

**Results:**

There were 116 (73.7%) survivors and 44 (26.3%) non-survivors. The rates of septic shock, norepinephrine administration, renal replacement, mechanical ventilator therapy, and reoperation, the Simplified Acute Physiology Score-3 (SAPS3), and the Sepsis-related Organ Failure Assessment (SOFA) score were greater in non-survivors. DNI on day 3 was better than the other laboratory variables for predicting mortality. DNI was correlated with the SAPS3 (*r* = .46, *p* = .00) and SOFA score (*r* = .45, *p* = .00). The optimal cut-off DNI for predicting mortality was 7.8% (sensitivity: 77.3%; specificity: 95.9%). In receiver-operating characteristic curve analysis, DNI on day 3 was the best indicator of mortality (area under the curve: .880; 95% confidence interval: .80–.96).

**Conclusions:**

Our results indicate that DNI is better than other laboratory variables for predicting postoperative mortality in patients with sepsis caused by peritonitis. DNI > 7.8% on day 3 was a reliable predictor of postoperative mortality.

## Introduction

Sepsis is one of the most common causes of death following surgery. Despite recent advances in antibiotics and general critical care practices, the mortality rate due to severe sepsis and septic shock is increasing worldwide.[1–3] Surgical site infection is a particularly serious problem that can delay recovery after surgery, increase hospital stay, and increase medical expenditure. The mortality rate due to sepsis was reported to range from 20% to 30%, and early recognition and risk stratification are necessary to improve the outcomes of patients with sepsis[4, 5].

Many investigators have searched for reliable biomarkers to aid the diagnosis and management of sepsis. The two most frequently used biomarkers are C-reactive protein (CRP) and procalcitonin, although they have limited value as diagnostic and prognostic markers.[6] To date, no single biologic marker has been shown to reliably identify patients who are at risk of developing severe sepsis or septic shock.[7, 8] Several clinical scores have been developed to assess disease severity and predict the outcome of sepsis that included the Acute Physiology and Chronic Health Evaluation (APACHE) score, Sequential Organ Failure Assessment (SOFA) score, and Simplified Acute Physiology Score (SAPS).

During infection, immature neutrophils enter the circulation. This “left-shift” response to infection is defined as an elevated ratio of immature granulocytes to total granulocytes.[9] Although it can be a useful marker of infection in clinical practice, modern automated cell analyzers can provide information on leukocyte differentials based on the nuclear lobularity of white blood cells (WBC) and the cytochemical myeloperoxidase (MPO) reaction.[10, 11] The delta neutrophil index (DNI), calculated as the difference between the leukocyte differential in the MPO channel and the leukocyte differential in the nuclear lobularity channel, was significantly associated with disseminated intravascular coagulation scores, the positive blood culture rate, and the mortality rate in patients with suspected sepsis.[10] Moreover, some studies have reported that the DNI is a more useful marker than the WBC count and CRP for predicting mortality in patients with sepsis.[12–14] However, these studies mostly comprised patients who underwent nonsurgical treatment.

Therefore, the aim of this study was to evaluate the usefulness of DNI for predicting postoperative mortality in patients with sepsis caused by peritonitis by means of comparing the DNI, WBC count, neutrophil percentage, and CRP before and after surgery.

## Materials and methods

### Patients and characteristics

This retrospective, observational study was performed at Kangdong Sacred Heart Hospital and Dongtan Sacred Heart Hospital, which are 600-bed teaching hospitals in Seoul, Korea. This study was approved by the institutional review board at Kangdong Sacred Heart Hospital (Ref. 2016-10-022-001). All data were fully anonymized before access and IRB waived the requirement for informed consent.

Patients who underwent surgery to treat sepsis caused by peritonitis between July 2011 and May 2016 were included in this study. Sepsis was defined according to the new consensus with diagnosis based on the combination of infection and SOFA score ≥ 2 points. Septic shock can be identified with a clinical construct of sepsis with persisting hypotension requiring vasopressors to maintain MAP ≥65 mm Hg and having a serum lactate level >2 mmol/L (18 mg/dL) despite adequate volume resuscitation.[9] Patients aged < 18 years, pregnant women, patients with hematologic abnormalities, and patients who received granulocyte colony stimulating factors, glucocorticoid, or other immunosuppressants were excluded from the study.

This study included a total of 160 patients, of whom 95 were recruited from the Kangdong Sacred Heart Hospital and 65 from the Dongtan Sacred Heart Hospital; their clinical characteristics are listed in Table 1. There were 116 (72.5%) survivors and 44 (27.5%) non-survivors. The rates of septic shock, norepinephrine administration, reoperation, renal replacement and mechanical ventilator therapy, and the ASA classification, SAPS3, and SOFA score were greater in non-survivors than in survivors.

**Table 1:** Clinical characteristics of survivors and non-survivors.

Variables	Total (n = 160, 100.0%))	Survivors (n = 116, 72.5%)	Non-survivors (n = 44, 27.5%)	*P* value
Age (years)	70 (59–79)	68 (58–80)	70 (60–76)	.869
Gender (male)	96 (60.0%)	69 (59.5%)	27 (61.4%)	.859
BMI (kg/m^2^)	22.3 (19.8–24.2)	22.2 (20.1–24.5)	20.8 (19.2–23.9)	.438
Septic shock	62 (38.8%)	25 (21.6%)	37 (84.1%)	< .001
Norepinephrine therapy	40 (25.0%)	10 (8.6%)	30 (68.2%)	< .001
Surgical site				.732
Small bowel	51 (31.9%)	38 (32.8%)	13 (29.5%)	
Colon	42 (26.3%)	32 (27.6%)	10 (22.7%)	
Gastroduodenal tract	24 (15.0%)	17 (14.7%)	7 (15.9%)	
Biliary tract	24 (15.0%)	16 (13.8%)	8 (18.2%)	
Other	12 (7.5%)	7 (6.0%)	5 (11.4%)	
Appendix	7 (4.4%)	6 (5.2%)	1 (2.3%)	
Reoperation	21 (13.1%)	11 (9.5%)	10 (22.7%)	.031
Operation time (min)	145(105–192)	147(110–193)	145(95–201)	.945
Positive blood culture	42 (26.3%)	29 (25.0%)	13 (29.5%)	.231
Gram negative	22 (13.8%)	15 (12.9%)	7 (15.9%)	
Gram positive	16 (10.0%)	12 (10.3%)	4 (9.1%)	
Fungus	5 (3.1%)	3 (2.6%)	2 (4.5%)	
Mechanical ventilation	112 (70.0%)	70 (60.3%)	42 (95.5%)	< .001
Renal replacement therapy	20 (12.5%)	10 (8.6%)	10 (22.7%)	.020
ASA physical status score	2.66± 0.81	2.57± 0.73	2.89± 0.97	.026
SAPS3	68 (55–79)	63 (51–71)	84 (74–97)	< .001
SOFA score	6 (4–10)	5 (3–8)	11 (8–13)	< .001
WBC day 3 (10^9^/L)	10.0 (7.2–13.3)	9.5 (6.9–12.4)	13.2 (7.5–16.9)	.023
Neutrophil day 3 (%)	84.3 (77.7–89.4)	83.1 (77.4–88.6)	87.6 (80.3–91.5)	.045
CRP day 3 (mg/L)	125.8 (75.6–183.9)	121.4 (75.6–166.7)	153.2(70.2–241.0)	.024
DNI day 3 (%)	2.8 (1.2–7.4)	2.1 (1.0–3.7)	15.9(8.1–39.8)	< .001

Values are expressed as the median (interquartile range) or number of patients (percent).

ASA physical status score is expressed as mean±standard deviation.

BMI, Body mass index; ASA, American Society of Anesthesiologists; SAPS, Simplified Acute Physiology Score; SOFA, Sequential Organ Failure Assessment; WBC, white blood cell; CRP, C-reactive protein; DNI, delta neutrophil index.

### Data collection

Patient data, including age, gender, diagnosis, operation time, transfusion, blood culture results, presence of septic shock, and hospital mortality were retrieved from medical records. In-hospital mortality was defined as death within 30 days of surgery for sepsis. We also retrieved data regarding reoperation, mechanical ventilation, and renal replacement therapy. The SAPS3[15] and SOFA score [11] were calculated to measure the severity of sepsis. The American Society of Anesthesiologists Physical Status classification system (ASA classification)[16] was recorded by the attending anesthesiologist.

### DNI and laboratory tests

Laboratory tests, which included DNI, WBC count, neutrophil percentage, and CRP, were measured within 12 h before surgery (day 0), at 12–36 h after surgery (day 1), and 60–84 h after surgery (day 3). DNI is routinely recorded in the complete blood count tests at our institution. DNI was determined using an automatic cell analyzer (ADIVA 2120 Hematology System; Siemens Healthcare Diagnostics, Forchheim, Germany). After red blood cell lysis, the cell size and stain intensity were measured using the tungsten-halogen-based optical system of the MPO channel to count and differentiate granulocytes, lymphocytes, and monocytes based on their size and MPO content. This was followed by cell counting and classification according to size, lobularity, and nuclear density, using the laser diode-based optical system of the lobularity nuclear density channel. The DNI was calculated as the neutrophil and the eosinophil subfraction measured in the MPO channel minus the polymorphonuclear neutrophil (PMN) subfraction measured in the nuclear lobularity channel, as previously described.[10]

### Statistical analysis

Statistical analyses were performed using SPSS 19.0 (IBM Corp., Armonk, NY, USA). Continuous variables are presented as the median (interquartile range) and categorical variables as the absolute number and percentage. As the normality test, a skewness and kurtosis test was used. Between-group comparisons were made using χ^2^ tests for categorical variables and independent-samples *t* tests for continuous variables. We used binary logistic regression analysis to assess the mortality rate according to the WBC count, neutrophil percentage, CRP, and DNI. The correlation between the DNI and clinical severity scores was tested using Pearson’s correlation analysis. The strength of the correlation was described using the guide that Evans suggests for absolute value of *r*. [17] Receiver-operating characteristic (ROC) curves were plotted and the Youden Index method was used to determine the optimal cut-off values for DNI, WBC count, neutrophil percentage, and CRP for predicting mortality. The area under the curve (AUC) was calculated to compare the performance of each marker for predicting mortality. In all analyses, a *p*-value of < .05 was considered statistically significant.

## Results

### Comparison of DNI and other laboratory markers between survivors and non-survivors

**Fig 1:**
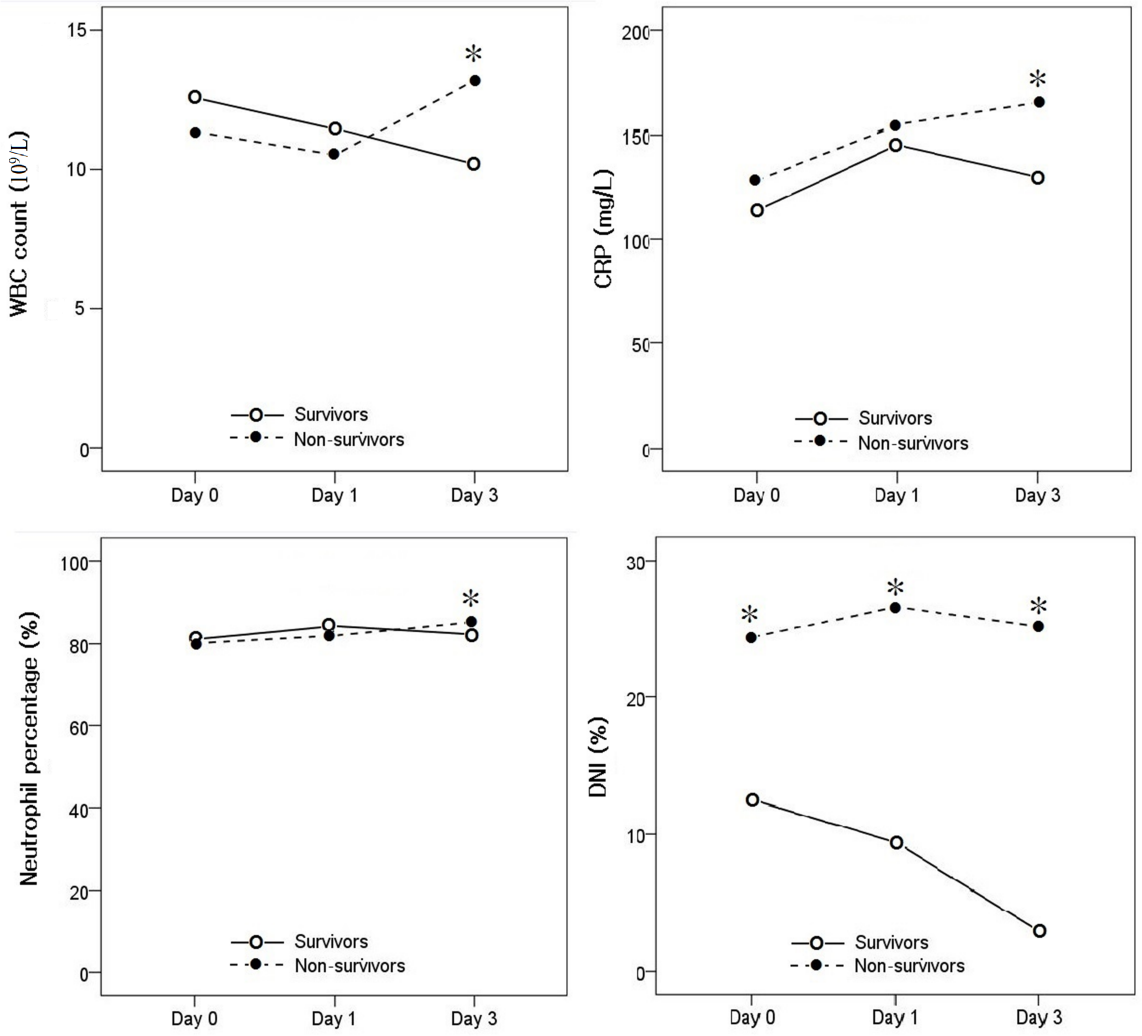
Comparison of changes in WBC count, neutrophil percentage, CRP, and DNI between the survivors and non-survivors. *CRP*, C-reactive protein; *DNI*, delta neutrophil index; *WBC*, white blood cell. *p < .05 vs. survivors.

The WBC count and neutrophil percentage on days 0 and 1 tended to be lower in non-survivors than in survivors, but the differences were not statistically significant. On day 3, there were statistically significant differences in the WBC count, neutrophil percentage, and CRP between the two groups(S1 Table). The DNI was significantly greater in non-survivors than in non-survivors before surgery and on days 1 and 3 after surgery (Fig 1). Logistic regression analysis was performed using data collected on day 3 when there were statistically significant differences in the WBC count, neutrophil percentage, CRP, and DNI. In this analysis, DNI was associated with postoperative mortality, with an odds ratio of 1.286 (*p* < .01) (Table 2).

**Table 2:** Logistic regression analysis of laboratory variables on postoperative day 3 for predicting postoperative mortality.

	OR	95% CI	SE	Wald statistic	*p*
WBC count	1.000	1.000–1.000	0.000	0.098	0.754
Neutrophil percentage	1.061	0.983–1.146	0.039	2.287	0.130
CRP	1.000	0.993–1.006	0.003	0.001	0.978
DNI	1.286	1.145–1.444	0.059	17.982	<0 .001

OR, Odds ratio; CI, confidence interval; SE, standard error; WBC, white blood cell; CRP, C-reactive protein; DNI, delta neutrophil index

Correct Classification Rate: 90.4%, Cox&Snell's R2 = .425, Nagelkerke's R2 = .621, Hosmer-Lemenshow x2 = 16.627(df = 8), *p*>0.05

### Relationship between DNI and clinical severity scores

**Fig 2:**
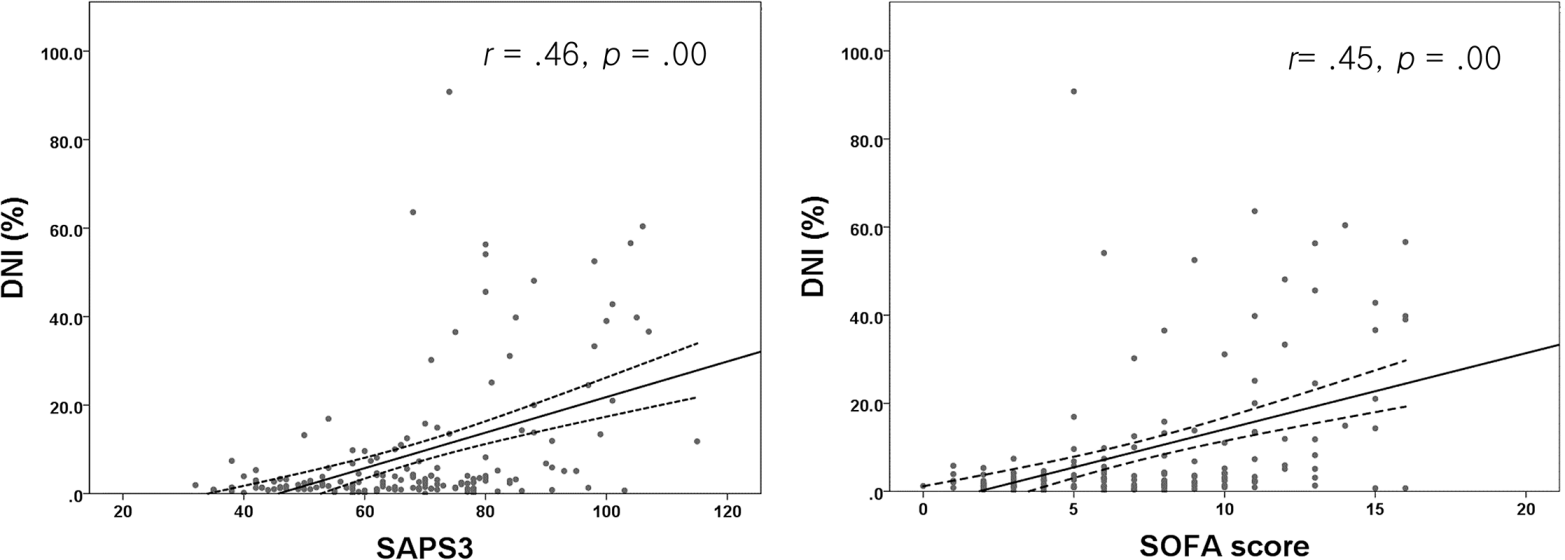
Correlation between clinical severity scores and the DNI on day 3 (60–84 h) after surgery. DNI, Delta neutrophil index; SAPS3, Simplified Acute Physiology Score-3; SOFA, Sepsis-related Organ Failure Assessment.

DNI was positively correlated with the SAPS3 (*r* = .46, *p* < .001, 95% confidence interval [CI]: 280–.524) and SOFA score (*r* = .45, *p* < .001, 95% confidence interval [CI]: 273 –.531) (Fig 2). The *r* values of DNI and clinical severity scores (SAPS3, SOFA score) were 0.45 and 0.46, which could be interpreted as positive moderate correlations.

### Performance of DNI and other laboratory markers for predicting mortality

**Fig 3:**
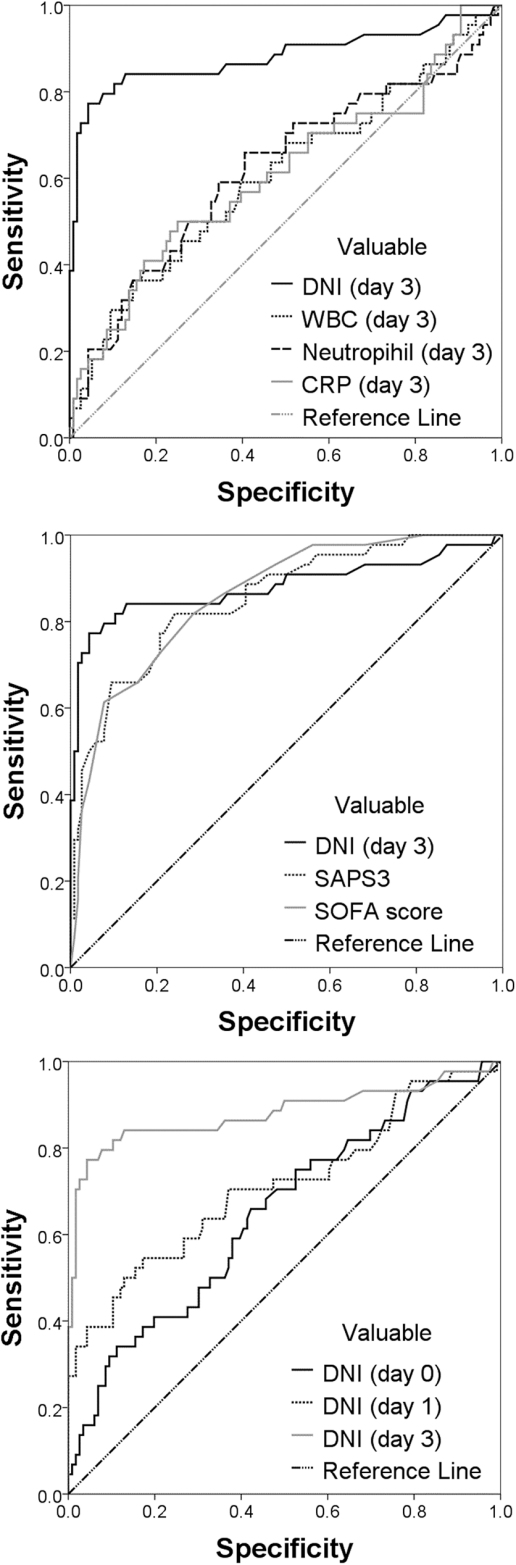
Receiver-operating characteristic curves for differentiating between survivors and non-survivors. CRP, C-reactive protein; DNI, delta neutrophil index; SAPS3, Simplified Acute Physiology Score-3; SOFA, Sepsis-related Organ Failure Assessment; WBC, white blood cell.

To obtain the optimal DNI cut-off value to predict mortality, we performed ROC curve analysis and calculated the AUC. As shown in Fig 3, the ROC curves revealed that the DNI on day 3 showed better performance for predicting mortality than did the WBC count, neutrophil percentage, and CRP, with an AUC of .88 (95% confidence interval [CI]: .804–.957). The AUCs for SAPS3 and SOFA score were both .85. When the DNI was analyzed for each measurement time individually, the AUCs were .65, .71, and .88 for the DNI on days 0, 1, and 3, respectively. The optimal cut-off DNI for predicting mortality was 7.8%, with a sensitivity of 77.3% and specificity of 95.9% (Table 3).

**Table 3:** Performance of laboratory markers for differentiating between survivors and non-survivors determined by receiver characteristic curve analysis.

	Cut-off value	Sensitivity (%)	Specificity (%)	AUC	95% CI
DNI	7.8 (%)	77.3%	95.7%	0.880	0.804–0.957
WBC count	14.1 (10^9^/L)	36.4%	85.3%	0.606	0.502–0.709
Neutrophil percentage	84.9 (%)	65.9%	59.5%	0.621	0.516–0.726
CRP	165.8 (mg/L)	50.0%	75.0%	0.609	0.504–0.714

DNI, delta neutrophil index; WBC, white blood cell; CRP, C-reactive protein; AUC, area under curve; CI, confidence interval

## Discussion

Sepsis is a leading cause of death in critically ill patients and its prevalence is increasing globally every year.[18–20] Early detection and early treatment of sepsis are essential to improve the patient’s outcome. Biomarkers such as CRP, procalcitonin, and various cytokines are elevated in sepsis. Therefore, these biomarkers are sometimes used as prognostic and diagnostic markers in patients with sepsis. There is a need to find more suitable and specific biomarkers for sepsis. The DNI is a known marker of infection, but many studies are still under way to evaluate its clinical usefulness in sepsis. Our data suggest that DNI measured at 60–84 h after surgical treatment of sepsis caused by peritonitis is a predictor of postoperative mortality.

The reported mortality rate in patients with sepsis caused by peritonitis ranges from 30%–50%,[21, 22] which is similar to the results in our study of a carefully selected cohort of patients. We also found that the rates of septic shock, norepinephrine administration, renal replacement, and mechanical ventilator therapy, and the ASA score, SAPS3, and SOFA score were greater in non-survivors than in survivors. These results are consistent with those of prior studies of sepsis.[12, 23] It is also intriguing that the AUCs for the SAPS3 and SOFA score (both .86) were similar to the AUC for the DNI (.88). Despite these similarities, the SAPS3 and SOFA score are calculated using distinct, complicated formulas. In our study, the optimal cut-off DNI for predicting mortality was 7.8%. Similarly, a previous study by Kim et al[13] reported that the optimal cut-off DNI for predicting mortality was 7.6% in patients with gram negative bacteremia. Meanwhile, Park et al[12] reported that a DNI > 6.5% was a good predictor of severe sepsis and septic shock within 24 h of admission to an intensive care unit. However, neither of those studies involved surgical patients. In this study, we retrieved the DNI, WBC count, neutrophil percentage, and CRP data from the medical records of patients who underwent surgery for peritonitis. These factors were measured before surgery and daily after surgery. On postsurgical day 3 (60–84 h), the WBC count, neutrophil percentage, and CRP were statistically significant predictors of mortality. For the DNI, the values on each measurement day were significant predictors of mortality, and the predictive value was greatest on day 3. Kim et al[13] reported that, in patients with gram negative bacteremia, the risk of early mortality was greatest in patients whose DNI remained higher than the initial value until 3 days after the onset of bacteremia. Lee et al[24] reported that a cut-off DNI of 2% at 72 h after the onset of neonatal sepsis was associated with the 7-day mortality rate. However, these studies involved nonsurgical treatment. Olivier et al[25] previously reported that the accuracy of CRP and procalcitonin as a predictor was greatest on postoperative day 4 in patients with colorectal cancer. Similar to that study, we reported that the DNI on postoperative day 3 was the best predictor of mortality in patients with sepsis caused by peritonitis. These results suggest that the DNI on postoperative day 3 accurately reflects the effect of surgical treatment on the progression of sepsis and patients who maintain a high DNI until 3 days after surgery have a poor prognosis; therefore, DNI on day 3 could be an alarm to check patients’ status once more and to consider other treatment strategies. In this study, we examined the correlation between DNI and postoperative mortality in patients with peritonitis-induced sepsis at different time points following surgery for the first time. Using DNI for postsurgery patients represents a novel approach because several previous studies focused on comparing changes in CRP and procalcitonin levels in nonsurgical patients at different time points. This study also demonstrated the correlation with clinical scores such as SOFA and SAPS 3, which measure the severity of illness and the outcome of sepsis.

Our study had several limitations. In particular, this was a retrospective review of medical records. For this reason, we could only evaluate data for the WBC count, neutrophil percentage, and CRP, which are routinely measured at our hospitals, whereas other biological markers, such as procalcitonin, lactic acid, and Disseminated Intravascular Coagulopathy score are not routinely assessed and could not be analyzed in this study. Because the blood samples for laboratory testing are routinely obtained from patients at around 7 a.m. at our hospitals, the blood samples taken on days 0, 1, and 3 were collected < 12 h before surgery, 12–36 h after surgery, and 60–84 h after surgery. The wide time intervals inevitably led to large gaps between the measurements. There are two limitations regarding statistics. First, the laboratory variables for predicting postoperative mortality were analyzed by the logistic regression instead of the binominal regression in Table 2. It may have affected the result according to the prevalence of the event. However, these laboratory variables for predicting mortality were also compared by other methods. The second limitation is about the normality test for data. In this study, skewness and kurtosis were used for the normality test. This may be a weak point of this study, but there are several papers arguing that there is no problem in testing for the normality using skewness and kurtosis.[26–30] Finally, because of the small sample size in this study, additional studies with large numbers of patients are needed to validate the clinical usefulness of the DNI as a predictor of postoperative mortality in patients with sepsis caused by peritonitis.

Despite these limitations, our results demonstrate that the DNI is a useful marker for predicting mortality of septic patients who underwent surgical treatment of peritonitis. The DNI is increasingly being performed as a routine laboratory test, along with complete blood count, and does not require any additional time or cost in clinical settings, unlike other biomarkers such as CRP and procalcitonin. The time and cost-saving feature of DNI make it advantageous in this setting. Prospective, multicenter studies with large numbers of patients are needed to validate our findings that the DNI is a promising marker for predicting the prognosis after peritonitis surgery and whether it should be introduced into the clinical guidelines for the surgical management of sepsis.

## Conclusion

Our study found that DNI, which reflects the proportions of immature granulocytes in circulating blood, was more accurate than the WBC count, neutrophil percentage, and CRP for predicting mortality of patients undergoing surgical treatment of sepsis caused by peritonitis. In particular, a DNI > 7.8% at 60–84 h after surgery was a strong predictor of postoperative mortality. Therefore, these findings suggest that the DNI may be a useful marker in the management of patients undergoing surgical treatment of sepsis caused by peritonitis.

## Supporting information

S1 Table Comparison of WBC count, neutrophil percentage, CRP, DNI, SAPS3 and SOFA score between the survivors and non-survivors. (DOCX)
