# Supplementary material for: The delta neutrophil index is a prognostic factor for postoperative mortality in patients with sepsis caused by peritonitis
Source: PLoS One. 2017 Aug 1;12(8):e0182325. doi: 10.1371/journal.pone.0182325 (PMC5538749; doi:10.1371/journal.pone.0182325)
Supplement: S1 Table — (DOCX) [file pone.0182325.s001.docx]

**S1 Table.** Comparison of WBC count, neutrophil percentage, CRP, DNI, SAPS3 and SOFA score between the survivors and non-survivors.

|  | Survivors  (n = 116, 72.5%) | Non-survivors  (n = 44, 27.5%) | t value | p value |
| --- | --- | --- | --- | --- |
| WBC day 0 (10^3^/mm^3^) | 12.7 ± 8.6 | 11.3 ± 9.1 | 0.862 | 0.390 |
| WBC day 1 (103/mm3) | 11.5 ± 7.7 | 10.5 ± 8.3 | 0.737 | 0.462 |
| WBC day 3 (103/mm3) | 10.2 ± 4.8 | 13.2 ± 7.9 | -2.335 | 0.023 |
| Neutrophil percentage day 0 (%) | 81.3 ± 11.7 | 80.1 ± 14.2 | 0.541 | 0.589 |
| Neutrophil percentage day 1 (%) | 84.7 ± 9.7 | 81.9 ± 12.0 | 1.495 | 0.137 |
| Neutrophil percentage day 3 (%) | 82.3 ± 8.1 | 85.2 ± 9.1 | -2.1935 | 0.045 |
| CRP day0 (mg/L) | 114.8 ± 92.1 | 128.6 ± 95.6 | -0.835 | 0.405 |
| CRP day1 (mg/L) | 145.5 ± 91.7 | 154.6 ± 93.6 | -0.557 | 0.578 |
| CRP day3 (mg/L) | 127.6 ± 73.5 | 164.9 ±96.7 | -2.32 | 0.024 |
| Delta neutrophil index day 0 (%) | 13.0 ± 7.5 | 24.3 ± 14.2 | -2.884 | < .001 |
| Delta neutrophil index day 1 (%) | 9.4 ± .1 | 26.6 ± 14.4 | -4.515 | < .001 |
| Delta neutrophil index day 3 (%) | 2.9 ± 2.5 | 25.2 ± 15.6 | -6.804 | < .001 |
| SAPS3 | 62.1 ± 14.3 | 84.2 ± 15.2 | -8.614 | < .001 |
| SOFA score | 5.7 ± 3.3 | 10.8 ± 3.3 | -8.362 | < .001 |

WBC, white blood cell; CRP, C-reactive protein; DNI, delta neutrophil index; SAPS, Simplified Acute Physiology Score; SOFA, Sequential Organ Failure Assessment; WBC, white blood cell; CRP, C-reactive protein; DNI, delta neutrophil index.
